# A Questionnaire Study to Assess the Belief and Barrier to Blood Donation and the Influence of Educational Intervention on Urban and Rural Patients

**DOI:** 10.7759/cureus.42520

**Published:** 2023-07-26

**Authors:** Monika Kumari, Irfan Ali, Binod Shankar, Mohnish Muchhal, Ambar Khan, Anmol Razdan

**Affiliations:** 1 Public Health Dentistry, Divya Jyoti College of Dental Sciences and Research, Modinagar, Ghaziabad, IND; 2 Public Health Dentistry, Divya Jyoti College of Dental Sciences and Research, Modianagar, Ghaziabad, IND; 3 Prosthodontics and Crown and Bridge, Hazaribag College of Dental Sciences and Hospital, Hazaribag, IND; 4 Oral Medicine and Radiology, I.T.S. College of Dental Education and Research College, Muradnagar, Ghaziabad, IND

**Keywords:** patients, motivation, life, disease, blood

## Abstract

Objective: Blood transfusion services are an essential part of any healthcare delivery system in today's clinical world with advanced medicines because blood is in high demand during various medical emergencies. Improving knowledge and hastening the development of a positive attitude toward blood donation in society should be the goal of developing an efficient strategy for sustaining a safe and adequate blood supply.

Aim: The study aims to explore the knowledge, attitude, and motivation toward blood donation among urban and rural patients attending the outreach program.

Materials and Methods: Patients who attended the outreach program held by the Dental College of Uttar Pradesh were subjected to a questionnaire survey to determine their level of knowledge regarding blood donation. Further, an education-based intervention was done among the camp patients to assess the change in their learning after the intervention. The difference between the individual responses prior to and following the intervention was analyzed using the chi-square test. p≤0.05 was considered statistically significant.

Results: Urban patients comprise 186 males (44.28%) and 234 females (55.72%). Among the rural patients, 205 (45.55%) were males and 245 were females (54.45%). About 48.80% of the urban patients and 48.88% of the rural patients were Hindus while the remaining were Muslims. Only 22.14% of urban patients and 16.88% of rural patients had donated blood till now. The comparison after the intervention for rural patients showed a significant improvement in the level of knowledge and awareness.

Conclusion: Although it is quite common knowledge that rural people require more awareness and education related to health, after the conduction of our study, we have resolute evidence that demands thorough IEC and health information activities for the welfare of rural people and to diminish the divide.

## Introduction

The human body is considered to be one of the amazing factories that makes the blood which is universally recognized as the most precious element that sustains life [1-2]. During various life-threatening conditions, blood donation can save the lives of many people, and saving the life of any person is one of the noblest causes [3]. But still, people died due to the unavailability of blood transfusion services during the “golden hour of first aid” [4]. According to World Health Organization, the blood donation rate in a high-income country is 33.1 donations per 1000 people, 11.7 donations per 1000 people in middle-income countries, and a low of 4.6 donations per 1000 people in low-income countries [5]. Blood transfusion services are an important part of any healthcare delivery system in today's clinical world with advanced medicines because there is a high demand for blood during various medical emergencies such as road traffic accidents, burns, hematological diseases, or major surgical interventions such as open heart and renal surgeries. By one blood donor, three lives can be saved at one time and it can only ensue from generous donors [6].

Every year on June 14th, World Blood Donor Day is celebrated to raise awareness about the importance of blood donation, and the need for safe blood and blood products, and to thank voluntary unpaid blood donors for their life-saving gifts of blood [6-7]. World Health Organization (WHO) gives a theme for raising awareness regarding blood donation benefits every year so that the maximum number of the population can take the best advantage of it, the theme for 2019 was “Blood donation and universal access to safe blood transfusion” and the slogan for the campaign was “Safe blood for all.” The World Health Organization (WHO) policy is to achieve 100% not-paid blood donation practice in 2020 [8].

According to WHO, yearly a total of 81 million units of blood are collected, but only 39% are collected in developing countries like South African and South East Asian countries which have 82% of the world’s population [9]. Obstetric bleeding and anemia were found to be responsible for 15%-20% of child mortality, as well as a quarter million maternal deaths in Africa because of the lack of blood availability [10].

With a population of approximately 120 million people, India is naturally a country that requires a large amount of blood to save citizens' lives. According to reports, our country requires approximately 8 million units of blood per year. Only half of this, or approximately 4 million units of blood, can be obtained from voluntary blood donors [6, 11-13]. India has only a total of 2,760 (government and private) blood banks which accounts for almost 46.5 lakh Indians per blood bank depicting the massive shortage of blood banks across the country. This answer is appalling as even now 64 out of 675 districts in the country do not have a single blood bank, neither under public nor under private operations [14]. 

Blood donations in India are conducted by several organizations and hospitals by organizing blood donation camps both governmental and non-governmental like Rotary Blood Bank, Indian Red Cross Society, Khoon Organization, Sankalp India Foundation, Save Life India, Lions Blood Bank, Think Foundation, Athar Blood Bank, HEROES, etc. Student organizations like the National Service Scheme (NSS), National Cadet Corps (NCC), Youth Red Cross (YRC), Students Green Clubs (SGC), and Red Ribbon Club (RRC) are a few well-known ones that have demonstrated their work in significant community-oriented services and uplifts aside from governmental and non-governmental organizations [15].

The attitude of a person toward blood donation differs between donors and non-donors. Altruism is one of the major and most commonly reported reasons motivating a subject to donate. The other positive attitudes associated with one’s decision to donate blood included national duty, religious acts, quality of service at the blood bank, and family needs or encouragement. One of the major negative attitudes reported by non-donors was fear [16-17]. The other barriers to blood donation that influence the behavior of people are cultural beliefs in some ethnic groups, socio-economic factors, and lack of knowledge [12]. In developing countries, only 60% of the people have adequate knowledge of blood donation [8, 18]. The objective in developing an effective strategy for sustaining a safe and adequate blood supply should be to increase knowledge and hasten the development of a positive attitude toward blood donation in society. The first step in achieving this goal should be to conduct an unbiased, exhaustive, and thorough assessment of societal knowledge and attitudes regarding blood donation [2, 10].

No such study has been done yet to assess the knowledge, attitude, and beliefs about blood donation among pharmacy students in India. Further, there is very limited literature available on the knowledge and belief of the general rural population regarding blood donation and the impact of educational intervention on improvement in their knowledge. Thus, the objective of the present study was to examine how the urban population and the rural population participating in the outreach program feel about blood donation and to determine the effects of educational intervention on knowledge gains (subjects attending the outreach program).

## Materials and methods

From June to August 2019, researchers at a dental college in Uttar Pradesh conducted an intervention-based survey using a self-structured questionnaire. The study included 520 patients from both urban and rural areas who attended screening/treatment camps run by the college between June 2019 and August 2019. Patients with a reported intellectual disability such as autism, dementia, mental retardation, etc. were excluded from the study and patients who gave consent were included in the study. The ethical clearance for the study was obtained from the institution review board of the college.

Questionnaire

The study was conducted in the Department of Public Health Dentistry at D.J College of Dental Sciences and Research, Modinagar where a self-administered questionnaire was distributed among the study participants. The study objectives, design, and questions were explained to all the participants who gave their consent and they were requested to complete the questionnaire within 15 min. A total of 420 urban patients (out of 460, 91.3% responded) and 450 rural patients (out of 520, 86.5% responded) participated in the study and returned the completed questionnaire.

For camp patients, a 10-item questionnaire was translated into the local language, Hindi, and validated using the test-re-test method (Cronbach's alpha value = 0.89). Then, during the camps run by the Department of Public Health Dentistry, this translated questionnaire was distributed and collected from the patients.

Once the questionnaire was collected from patients, an educational intervention was carried out for them by a senior faculty member of a Medical College which included a 20-min interaction and motivation sessions on blood donation. The educational component mainly concentrated on the materials that highlighted the laws, significance, and advantages of blood donation, as well as legal concerns and inspirational messages. The same questionnaire was again distributed and used to gather information from camp patients right after this session.

Data management and statistical analysis

The collected data were analyzed using a statistical package for social sciences (SPSS) 21.0 (SPSS Inc., Chicago, IL, USA). Chi square test was used to compare the individual responses of camp patients before and after the intervention. Mean difference in responses among the camp patients before and after intervention was assessed using an independent t-test. p≤0.05 was considered statistically significant.

## Results

The study included 420 urban and 450 rural patients who attended treatment camps. Table 1 shows the socio-demographic characteristics. The mean age of urban patients was 22.8 ± 13.6 and consisted of 186 males (44.28%) and 234 females (55.72%). Among the rural patients, the mean age was 24.5 ± 14.7, and consisted of 205 (45.55%) were males and 245 were females (54.45%). Only 22.14% of urban and 16.88% of rural patients had donated blood till now. Out of the 450 patients, 370 (80.7%) were literate and had at least completed their primary education. None of the patients had completed college or higher education.

**Table 1:** Demographic characteristics of the study subjects.

Demographic characteristics		Urban patients (420) n(%)	Rural patients (450) n(%)
Gender	Male	186 (44.28%)	205 (45.55%)
	Female	234 (55.72%)	245 (54.45%)
Religion	Hindu	205 (48.80%)	220 (48.88%)
	Muslim	215 (51.20%)	230 (51.12%)
Those who had previously donated blood		93 (22.14%)	76 (16.88%)

Table 2 highlights the responses to the questionnaire -- 30% of the camp population were not aware of the concept of blood donation; 77.34% patient population were not aware of the rules and legislation about blood donation; 39.05% of urban and 67.77% of rural patients feel that blood donation is against religion; 50.47% urban and 79.77% rural patients prefer donating blood to close relatives or known only; 25% urban patients’ family and 45.55% rural patients’ family were against the decision of blood donation; 74.28% urban and 72.45% rural patients think that there is a lack of information about blood donation in our country; 41.43% of urban patients and 78.23% rural patients fear that the blood donated is misused.

**Table 2:** Response to the questionnaire of respondents before the intervention.

Sl.No	Questions	Urban patients (420)		Rural patients (450)	
		Yes n(%)	No n(%)	Yes n(%)	No n(%)
1.	Are you aware of the concept of blood donation?	395 (94%)	25 (6%)	315 (70%)	135 (30%)
2.	Do you think blood donation is a national duty?	386 (91.90%)	34 (8.10%)	256 (56.88%)	194 (43.12%)
3.	Do you think that blood donation can be done easily every 6 months?	401 (95.47%)	19 (40.5%)	186 (41.33%)	264 (58.67%)
4.	Are you aware of the rules and legislation about blood donation?	281 (66.90%)	139 (33.10%)	102 (22.66%)	348 (77.34%)
5.	Do you feel that blood donation is against your religious beliefs?	164 (39.05%)	256 (60.95%)	305 (67.77%)	145 (32.23%)
6.	Do you prefer donating your blood to your close relatives/known to only?	212 (50.47%)	208 (49.53%)	359 (79.77%)	91 (20.23%)
7.	Do you think your family will support your decision on blood donation?	315 (75%)	105 (25%)	245 (54.45%)	205 (45.55%)
8.	Do you agree that there is a lack of information about blood donation in our country among the masses?	312 (74.28%)	108 (25.72%)	326 (72.45%)	124 (27.55%)
9.	Are you willing to educate the masses about the significance of blood donation?	389 (92.61%)	31 (7.39%)	278 (61.77%)	172 (38.23%)
10.	Do you fear that the blood donated by you may be misused?	174 (41.43%)	246 (58.57%)	352 (78.23%)	98 (21.77%)

Table 3 shows the comparison before and after intervention for camp patients, a significant improvement in the level of knowledge and awareness was observed. Following the intervention, approximately 75.55% of the population believe that blood donation is not against religious beliefs. After the educational intervention, there was also a positive change in patients' attitudes toward the religious barrier and family support.

**Table 3:** Response to the questionnaire of the patients before and after the intervention. The test used: Chi-square test **Statistically significant value (p ≤ 0.05)

Sl. No	Questions	Pre-Intervention		Post-Intervention		p Value
		Yes n(%)	No n(%)	Yes n(%)	No n(%)	
1.	Are you aware of the concept of blood donation?	315 (70%)	135 (30%)	430 (95.55%)	20 (4.45%)	<0.01**
2.	Do you think blood donation is a national duty?	256 (56.88%)	194 (43.12%)	410 (91.12%)	40 (8.88%)	0.04**
3.	Do you think that blood donation can be done easily every 6 months?	186 (41.33%)	264 (58.67%)	428 (95.12%)	22 (4.88%)	0.06
4.	Are you aware of the rules and legislation about blood donation?	102 (22.66%)	348 (77.34%)	415 (92.22%)	35 (7.78%)	0.04**
5.	Do you feel that blood donation is against your religious beliefs?	305 (67.77%)	145 (32.23%)	110 (24.45%)	340 (75.55%)	0.02**
6.	Do you prefer donating your blood to your close relatives/known to only?	359 (79.77%)	91 (20.23%)	140 (31.12%)	310 (68.88%)	0.05**
7.	Do you think your family will support your decision on blood donation?	245 (54.45%)	205 (45.55%)	290 (64.45%)	160 (35.55%)	0.01**
8.	Do you agree that there is a lack of information about blood donation in our country among the masses?	326 (72.45%)	124 (27.55%)	185 (41.12%)	265 (58.88%)	<0.01**
9.	Are you willing to educate the masses about the significance of blood donation?	278 (61.77%)	172 (38.23%)	315 (70%)	135 (30%)	0.08
10.	Do you fear that the blood donated by you may be misused?	352 (78.23%)	98 (21.77%)	329 (73.12%)	121 (26.88%)	<0.01**

The pre- and post-intervention responses with the mean and standard deviation were recorded in Table 4 using an independent t-test which show statistically significant results for all questions except the fear that blood donated by them may be misused.

**Table 4:** Mean response to the questionnaire of patients before and after intervention. The test used: independent t-test **Statistically significant value (p ≤ 0.05)

Sl.No	Questions	Mean ± SD		Mean difference	p Value
		Pre-intervention	Post-intervention		
1.	Are you aware of the concept of blood donation?	0.70 ± 0.459	0.96 ± 0.206	-0.256	0.001^**^
2.	Do you think blood donation is a national duty?	0.57 ± 0.496	0.91 ± 0.285	-0.342	0.001^**^
3.	Do you think that blood donation can be done easily every 6 months?	0.41 ± 0.493	0.95 ± 0.216	-0.538	0.001^**^
4.	Are you aware of the rules and legislation about blood donation?	0.23 ± 0.419	0.92 ± 0.268	-0.696	0.001^**^
5.	Do you feel that blood donation is against your religious beliefs?	0.68 ± 0.468	0.24 ± 0.430	0.433	0.001^**^
6.	Do you prefer donating your blood to your close relatives/known to only?	0.80 ± 0.402	0.31 ± 0.463	0.487	0.001^**^
7.	Do you think your family will support your decision on blood donation?	0.54 ± 0.499	0.64 ± 0.479	-0.100	0.002^**^
8.	Do you agree that there is a lack of information about blood donation in our country among the masses?	0.72 ± 0.447	0.41 ± 0.493	0.313	0.001^**^
9.	Are you willing to educate the masses about the significance of blood donation?	0.62 ± 0.486	0.70 ± 0.459	-0.082	0.009^**^
10.	Do you fear that the blood donated by you may be misused?	0.78 ± 0.413	0.73 ± 0.444	0.051	0.074

## Discussion

Blood donation can be a lifesaving procedure since blood is an essential element of human life and there are no substitutes for it. Most blood is collected from willing donors through outdoor blood donation camps that are organized in educational institutes, commercial, and industrial houses. But, blood unavailability is still a serious and rising issue in the area of transfusion. Knowledge and awareness about blood donation are very incomplete among potential donors. It is important to spread the message that a medical degree is not required for an average person to save a life; instead, donating blood can save three lives rather than just one [7, 9].

Studies that were done by Wilkinson and Gupta (2016) and Chauhan et al. (2018) analyzing the perception and awareness about blood donation among the different communities before and after the intervention may come out to be useful to understand the effect of such interventions which in turn helps the implementation of relevant donor recruitment [12-13]. Hence, the present study was conducted to explore the knowledge, attitude, and motivation toward blood donation and compare pre- and post-intervention among the local population.

The current study attempted to compare knowledge and awareness levels about blood donation among urban and rural patients. The findings revealed several characteristics of the respondents regarding their knowledge, attitude, and beliefs about blood donation before and after the educational intervention.

In the present study, only 22.14% of urban patients and 16.88% of rural patients had earlier donated blood in their lifetime. These results were in agreement with the studies by Amurdhavani (2016) and Wilkinson and Gupta (2016), where 21% of female students in Chennai and 14.5% of youth in Nagpur, respectively, had donated blood earlier [3, 12]. The studies by Singh et al. (28.5%), Melku et al. (12.5%), and Tadesse et al. (24.5%) also showed similar results in accordance with the present study for the percentage of respondents who had donated blood earlier [4, 18-19]. In this study, awareness of blood donation was seen in 94% of urban patients and 70% of rural patients. A similar observation was observed in the study by Singh et al., in 2015 on medical students and nursing students in Kolar that 85% of students were aware of the concept of blood donation [4]. In the study conducted by Wilkinson and Gupta in 2016, 86% of the respondents were aware of the concept of blood donation [12]. The current finding reflects the patients' suboptimal level of knowledge about blood donation, which in turn reflected the general public's attitude.

Around 91.90% of urban patients and 56.88% of rural patients in the present study felt that blood donation is a national duty of each individual and this result shows agreement with the study by Abolfotouh et al. (2014) in which the majority (82.6%) of the respondents agreed with the same [16].

It was found during the study, 95.47% of urban patients and 41.33% of rural patients were aware that blood donation can be easily done within every 6 months. This finding is similar to the results of Jemberu et al. (2016), who discovered that 53.8% of adults in Debre Markos town were aware that people can donate once every three months. A total of 66.90% of urban patients and 22.66% of rural patients were aware of the rules and legislation about blood donation. In a study by Jemberu et al. (2016), considering 31 knowledge questions, 56.5% of the participants scored mean and above which indicates they are knowledgeable about blood donation rules and regulations [10].

In the present study, 39.05% of urban patients and 67.77% of rural patients thought that blood donation was against their religious beliefs. Similarly, 58.7% of the participants in a study by Abolfotouh et al. (2014) did not consider blood donation as a religious duty. The present study showed 79.77% of rural patients preferred to donate blood to their close relatives and friends only. This finding was almost similar to a study conducted by Abolfotouh et al. (2014) in which 91% donate blood to their close relatives and friends [16]. But the studies conducted by Hiremath et al. in 2012 and Jemberu et al. in 2016 found that only 30% and 36%, respectively, donated blood to their close relatives and friends which contradicts the present study and the reason for this was study subjects were voluntary blood donors in these studies [6, 10].

In the present study, about 45.55% family did not support the decision of blood donation but this was in contrast with the study conducted by Wilkinson and Gupta in 2016 in which only 4.1% did not donate blood as their family did not allow them. The cause must be a lack of unawareness among both young and old individuals. This disparity could also be related to a fear of blood or needles, lack of education, cross-cultural variances in living standards, religious beliefs, and cultural myths about blood donation [12].

Around 41.43% of urban patients and 78.23% of rural patients feared that the donated blood may be misused. In contradiction, only 5.75% respondents in the study by Dubey et al. (2014) felt that their blood will be misused by blood banks [20].

Around 70% of rural patients after the intervention and 92.61% of urban patients showed willingness to educate the masses about significance of blood donation. In a study by Melku et al. (2018), 89% respondents were ready for encouraging other people to donate blood [18].

Around 74.28% of urban patients and 72.45% of rural patients agreed that there is a lack of public awareness about blood donation in our country. A statistically significant improvement in the level of knowledge and awareness was observed when the responses of rural patients to questionnaires before and after intervention were analyzed [20]. Thus, the study's findings revealed the need for a drastic strategy to be implemented in order to educate the community about the importance of blood donation.

As a limitation of this study, it was cross-sectional in nature, which posed problems in relation to hypothesis testing since data on risk factors and outcomes were assessed at the same time. Furthermore, the current study is a questionnaire-based study, another potential limitation is bias, such as participant bias or memory bias, which may affect the responses of the study participants, leading to misleading study results. The present study included a sample size of only 870 individuals (420 urban and 450 rural), which affect the generalizability of the results. Thus, the sample size of the study should be increased so that the results can be generalized. Hence, well-designed longitudinal interventional studies with a larger sample size are required to establish knowledge, attitude, and motivation toward blood donation among the rural and urban populations.

Thus, it is recommended to educate students regarding safe blood donation and should be included in the school curriculum. The value of safe blood donation, the demand and supply for blood, and other related topics could be covered in the school curriculum, thus helping raise awareness among people from a young age itself. At the university level, a provision for blood donation awareness programs, as well as blood donation drives, will undoubtedly increase the youth's awareness.

On the occasion of World Blood Donor Day on June 14, institutes across the nation carry out yearly blood donation drives to spread awareness among students. There are also some key interventions implemented by the State government, like regular blood donation camps at the Gram Panchayat level. This helped create a platform for individuals to donate blood conveniently and also raised awareness about the significance of blood donation within local communities. 

Aside from physical camps, social media has proven to be a useful technique for raising awareness. By leveraging social media platforms, information about upcoming camps and the importance of blood donation reached a wider audience, encouraging more individuals to participate. Active participation from NGOs and other agencies is also crucial to actively participate in creating awareness and promoting blood donation. Thus, through the implementation of various educational interventions such as regular blood donation drives, social media campaigns, and active participation from NGOs, we can bridge the gap between belief barriers for blood donation.

## Conclusions

The present study revealed a substantial knowledge gap amongst the patients for blood donation. It can also be concluded that majority of the study subjects had not donated blood till now and not aware about the concept of blood donation. Many governmental, non-governmental organizations (NGOs), and student groups are frequently working for conducting blood donation camps and motivating lectures across the country but still our country is facing the problem of life-threatening conditions due to deficiency of blood in blood banks. The positive influence of educational intervention in the current study emphasizes the need for an intervention that incorporates knowledge, motivational messages, facts, and figures to bring about necessary changes in the patients’ perceptions and intentions regarding blood donation, highlighting the importance of health education and health promotion. There is a need to educate and motivate people to be volunteer blood donors more effectively, and to use these volunteer donors to educate and encourage additional youth on a regular basis to effectively participate in blood donation. Blood donation is an urgent need, and immediate action is required to disseminate information about it in order to save so many lives. Blood donation awareness and motivation can influence one's willingness to donate blood, which could ultimately help to increase the number of voluntary blood donors.

## References

[1] Shidam UG, Lakshminarayanan S, Saurabh S (2015). Knowledge and attitude regarding blood donation in rural Puducherry, India. Natl J Commun Med.

[2] Kurian RN, Sarkar SA. (2016). Study to assess the knowledge and attitude regarding blood donation among the general public in a selected urban area of New Delhi. Int J Nurs Midwif Res.

[3] Amurdhavani BS (2016). Awareness of blood donation among female college students - a survey. J Pharm Sci Res.

[4] Singh S, Chandrappa M, Venkatesha M (2015). Blood donation awareness and beliefs among medical and nursing students. Int J Med Sci Public Health.

[5] Elnajeh M, Ghazi HF, Abdalqader MA (2017). Knowledge, attitude and practice towards blood donation and its associated factors among university students in Shah Alam, Malaysia. Int J Commun Med Public Health.

[6] Hiremath P (2012). To assess the knowledge of blood donation among voluntary blood donor at blood bank, Krishna Hospital Karad (Maharashtra, India). J Nurs Care.

[7] Marwaha N (2015). Voluntary blood donation in India: achievements, expectations and challenges. Asian J Transfus Sci.

[8] Gebresilase HW, Fite RO, Abeya SG (2017). Knowledge, attitude and practice of students towards blood donation in Arsi university and Adama science and technology university: a comparative cross sectional study. BMC Hematol.

[9] Unnikrishnan B, Rao P, Kumar N (2011). Profile of blood donors and reasons for deferral in coastal South India. Australas Med J.

[10] Jemberu YA, Esmael A, Ahmed KY (2016). Knowledge, attitude and practice towards blood donation and associated factors among adults in Debre Markos town, Northwest Ethiopia. BMC Hematol.

[11] Dhara A, Dinesh K (2012). A qualitative study of perceptions about voluntary blood donation among the supportive service employees of a multi-specialty rural tertiary care hospital. Nat J Commun Med.

[12] Wilkinson A, Gupta RS (2016). Perceptions of blood donation amongst the youth. Panacea J Med Sci.

[13] Chauhan R, Kumar R, Thakur S (2018). A study to assess the knowledge, attitude, and practices about blood donation among medical students of a medical college in North India. J Fam Med Prim Care.

[14] Saha S, Chandra B (2016). A cross-sectional blood study in India: from donation activities of donors to blood bank services. Curr Sci.

[15] Siromani U, Thasian T, Isaac R (2013). Shortage of blood! Youths, the trend setters, can they help to meet the needs?. Nat J Med Res.

[16] Abolfotouh MA, Al-Assiri MH, Al-Omani M (2014). Public awareness of blood donation in Central Saudi Arabia. Int J Gen Med.

[17] Sachdev S, Mittal K, Patidar G (2015). Risk factors for transfusion transmissible infections elicited on post donation counselling in blood donors: need to strengthen pre-donation counselling. Indian J Hematol Blood Transfus.

[18] Melku M, Asrie F, Shiferaw E (2018). Knowledge, attitude and practice regarding blood donation among graduating undergraduate health science students at the University of Gondar, Northwest Ethiopia. Ethiop J Health Sci.

[19] Tadesse W, Ayalew Y, Yisma E (2018). Knowledge, attitude, practice and associated factors towards voluntary blood donation among regular health science students of Samara University, Ethiopia. Health Sci J.

[20] Dubey A, Sonker A, Chaurasia R (2014). Knowledge, attitude and beliefs of people in North India regarding blood donation. Blood Transfus.

